# Bloodstream Infection Incidence of Different Central Venous Catheters in Neonates: A Descriptive Cohort Study

**DOI:** 10.3389/fped.2017.00142

**Published:** 2017-06-20

**Authors:** Gerdina H. Dubbink-Verheij, Vincent Bekker, Iris C. M. Pelsma, Erik W. van Zwet, Vivianne E. H. J. Smits-Wintjens, Sylke J. Steggerda, Arjan B. te Pas, Enrico Lopriore

**Affiliations:** ^1^Division of Neonatology, Department of Pediatrics, Leiden University Medical Center, Leiden, Netherlands; ^2^Department of Statistics, Leiden University Medical Center, Leiden, Netherlands

**Keywords:** central venous catheterization, infant, umbilical catheter, femoral venous catheter, peripherally inserted central catheter, central line-associated bloodstream infection

## Abstract

Central venous catheters (CVCs) in neonates are associated with a risk of central line-associated bloodstream infections (CLABSI). Most reports on the incidence of CLABSI in neonates focus on umbilical venous catheters (UVCs) and peripherally inserted central catheters (PICCs). We evaluated the incidence and risk factors for CLABSI in a cohort of neonates with femoral venous catheters (FVCs), UVCs, and PICCs, with a gestational age ≥34 weeks born between January 1, 2006 and June 30, 2013. We included 2,986 neonates with a total of 656 catheters. The CLABSI incidence rate varied from 12.3 per 1,000 catheter-days in FVCs to 10.6 per 1,000 catheter-days in UVCs and 5.3 per 1,000 catheter-days in PICCs. In a Kaplan–Meier survival analysis, we did not find a difference in CLABSI risk between the catheter types (*p* = 0.29). The following factors were independently associated with an increased risk of CLABSI: parenteral nutrition [hazard ratio (HR) 2.60, 95% confidence interval (CI) 1.25–5.41], male gender (HR 2.63, 95% CI 1.17–5.90), and higher birth weight (HR 1.04, 95% CI 1.002–1.09), whereas antibiotic treatment at birth (HR 0.25, 95% CI 0.12–0.52) was associated with a decreased risk. Conclusion: In our cohort, we did not find a difference between the CLABSI incidence in FVCs, PICCs, and UVCs. Occurrence of CLABSI is associated with parenteral nutrition, male gender, and higher birth weight. Antibiotic treatment at birth was associated with a decreased risk of CLABSI.

## Introduction

Central venous catheters (CVCs) are frequently used in critically ill neonates admitted to the neonatal intensive care unit (NICU). The most common CVCs in the NICU are umbilical venous catheters (UVCs) and peripherally inserted central catheters (PICCs) (1). Femoral venous catheters (FVCs) are less often used and less well studied in neonatal populations (2).

Although CVCs in neonates have many benefits, they have also important disadvantages. CVC use has been associated with an increased risk for developing hospital-acquired bloodstream infections, also termed central line-associated bloodstream infections (CLABSI) (3, 4).

Bloodstream infections in neonates are associated with longer stay in the hospital, unfavorable outcome, and mortality (5). Risk reduction of these infections is important to improve the outcome of neonates.

Bloodstream infections in neonates have been studied extensively (6), but little is known about the influence of the type of catheter used on the CLABSI incidence. Only a few studies compared the risk of CLABSI between the different types of catheters, mostly in small and heterogeneous cohorts of neonates (3, 7–12).

In this study, we evaluate the incidence of and potential risk factors associated with CLABSI comparing FVCs, UVCs, and PICCs in a cohort of term and late preterm neonates.

## Materials and Methods

### Design

A descriptive cohort study design was used. The study was conducted at the NICU of the Leiden University Medical Center, a tertiary care center in The Netherlands. The study was approved by the hospital system institutional review board. Written informed consent was deemed not necessary because the study participants were anonymized.

### Patient Characteristics

From January 1, 2006 to June 30, 2013, 2,986 neonates of 34 or more weeks’ gestation were admitted to our NICU. The threshold of 34 weeks gestational age was chosen because FVCs are not placed in neonates of lower gestational ages at our NICU. The medical charts of all neonates were reviewed to select neonates with catheter placement during admission. According to our protocol, CVCs were placed in all neonates who needed mechanical ventilation or circulatory support by inotropes. Other indications for a CVC at our unit are difficulties to get intravenous access and the need for an exchange transfusion. There were no protocol changes regarding the indication for CVCs during the study period. We excluded neonates with their catheters being removed within 12 h after placement and neonates with missing data on CLABSI occurrence or catheter use.

### Data Collection

Gestational age at birth, birth weight, and gender were recorded. In addition, respiratory distress syndrome (RDS), defined as the need for mechanical ventilation and surfactant, administration of parenteral nutrition during catheter dwell time, type of CVC, age at catheter insertion, duration of catheter dwell time, use of antibiotics during the first 24 h after birth, prophylactic heparin treatment during catheter use, indication for catheter removal, proven sepsis, and CLABSI including type of causative microorganism were recorded. Small for gestational age was defined as a birth weight adjusted for gestational age below the 10th centile according to growth curves for Dutch neonates (13). The choice to use a specific CVC type was left to the discretion of the care-giver. In general the first days after birth, a UVC was the first catheter to choose. In case of failure to place a UVC and in older neonates, a PICC or FVC was inserted. UVCs were placed in high position.

Proven sepsis was defined by a positive blood culture in the presence of clinical signs suggestive of infection. A positive blood culture without symptoms in the neonate was considered as contamination. CLABSI was defined as proven sepsis occurring with a catheter in place for more than 2 days or with a catheter, which was in place for more than 2 days and was removed within 48 h before the clinical onset of sepsis. The incidence of CLABSI was measured as the percentage of CLABSI in the group and as CLABSI events per 1,000 catheter-days.

Removal of the catheter was recorded either as elective or non-elective. A non-elective removal was defined as an unresolvable complication leading to removal of the catheter prior to the completion of therapy for which the catheter was placed.

### Statistical Analysis

Data are given as median with range unless otherwise mentioned. For comparison of continuous variables, the Kruskal–Wallis test was used. *Post hoc* comparisons were performed using Mann–Whitney *U* test. For initial and *post hoc* comparisons of categorical variables, we performed the Chi-square or Fisher’s exact test where appropriate. Upon initial testing, *p*-values below 0.05 were considered significant. In *post hoc* testing, we applied a Bonferroni adjustment to the alpha values to control for Type 1 errors. In this comparisons, *p*-values below 0.017 (0.05/3) were significant. To estimate survival or duration of stay of the different types of catheters, the Kaplan–Meier method was used. An event was defined as the occurrence of a central line infection. Patients were censored after removal of the central line or at transfer to another hospital. Cox regression analysis was performed to analyze risk factors contributing to time to CLABSI. We chose to analyze gestational age, gender, birth weight, RDS, type and amount of catheters placed, use of prophylactic heparin, use of antibiotics during the first 24 h after birth, and parenteral nutrition during catheter dwell time to search for possible risk factors. If *p*-values were below 0.20 in a univariate analysis, these variables were analyzed using a multivariate Cox regression model in which *p*-values below 0.05 were considered statistically significant. All statistical analyses were performed using SPSS 20 (IBM Software, Armonk, NY, USA).

## Results

### Patient Characteristics

A total of 2,986 neonates born at 34 or more weeks’ gestation were admitted to our NICU during the study period, of which in 21% (*n* = 628) one or more CVCs were placed. A total of 76 neonates were excluded because their catheter was removed within 12 h (*n* = 73) and data could not be retrieved (*n* = 3).

In the remaining 552 neonates, 656 CVCs were placed, including 407 (62%) UVCs, 185 (28%) PICCs, and 64 (10%) FVCs. In 466 neonates, one CVC was placed. The remaining neonates had two (*n* = 71), three (*n* = 12), or four (*n* = 3) CVCs. In 19 neonates, two catheters were in place simultaneously during a period of more than 1 day. Baseline patient characteristics are given in Table 1. Neonates with FVCs had a higher gestational age at birth and a higher birth weight compared to neonates with other types of catheters. The percentage of neonates with congenital heart disease was also different in the three catheter groups.

**Table 1:** Baseline patient characteristics for the total population and per catheter type.

Variable	Total population (*n* = 552)	Femoral venous catheter (FVC) (*n* = 64)	Umbilical venous catheter (UVC) (*n* = 407)	Peripherally inserted central catheter (PICC) (*n* = 185)	*p*-Value	FVC vs UVC	FVC vs PICC	UVC vs PICC
Gestational age at birth (weeks)	38 (34–42)	39 (34–42)	38 (34–42)	38 (34–42)	**0.03**	**0.007**	**0.04**	0.84
Birth weight (g)	3,155 (807–6,430)	3,411 (1,742–4,700)	3,115 (1,130–6,430)	3,020 (807–4,900)	**0.001**	**0.002**	**<0.001**	0.12
Male gender, % (*n*)	56 (309)	58 (37)	54 (220)	57 (105)	0.75			
SGA, % (*n*)	13 (74)	5 (3)	14 (55)	17 (31)	0.05			
RDS, % (*n*)	19 (105)	17 (11)	21 (85)	15 (27)	0.18			
CHD, % (*n*)	18 (102)	42 (27)	13 (54)	32 (59)	**<0.001**	**<0.001**	0.135	**<0.001**
Asphyxia, % (*n*)	9 (48)	9 (6)	10 (39)	5 (9)	0.15			
PDA, % (*n*)	1 (5)	2 (1)	0.7 (3)	1 (2)	0.78			
Pneumothorax, % (*n*)	4 (23)	5 (3)	4 (18)	5 (9)	0.96			
Mortality <1 month, % (*n*)	8 (42)	6 (4)	8 (33)	8 (14)	0.21			
Duration of hospitalization (days)	8 (1–62)	11 (2–53)	8 (1–49)	13 (2–62)	**<0.001**	**<0.001**	0.18	**<0.001**

*Data are represented as median (range) unless otherwise specified. Upon initial testing, *p*-values < 0.05 were considered significant. *Post hoc* testing was significant if *p*-values were <0.017. Significant *p*-values are depicted in bold*.

*SGA, small for gestational age, defined as a birth weight adjusted for gestational age below the 10th centile according to growth curves for Dutch neonates (13); RDS, respiratory distress syndrome, defined as the need for mechanical ventilation and surfactant; CHD, cyanotic congenital heart disease; asphyxia, asphyxia for which therapeutic cooling was indicated; PDA, persistent ductus arteriosus needing treatment (conservative or surgical)*.

### Catheter Characteristics and Duration of Stay of the Catheter

Catheter characteristics are shown in Table 2. Median length of catheter dwell time varied from 5 days in UVCs to 6 days in PICC, whereas median length of FVC dwell time was not significantly different from both other catheter types. Age at catheter insertion and catheter removal, percentage of parenteral nutrition during catheter dwell time, and percentage of antibiotic treatment within 24 h postpartum varied significantly between catheter location groups.

**Table 2:** Catheter characteristics by catheter type.

Variable	Femoral venous catheter (FVC) *(n* = *64)*	Umbilical venous catheter (UVC) *(n* = *407)*	Peripherally inserted central catheter (PICC) *(n* = *185)*	*p*-Value	FVC vs UVC	FVC vs PICC	UVC vs PICC
Total amount of catheter-days	405	2,166	1,324				
Amount of catheter-days	6 (1–19)	5 (1–22)	6 (1–31)	**0.001**	0.051	0.696	**0.001**
Age at catheter insertion	4.5 (1–50)	1 (1–13)	5 (1–39)	**<0.001**	**<0.001**	0.407	**<0.001**
Age at catheter removal	10 (3–55)	6 (1–22)	12 (2–45)	**<0.001**	**<0.001**	0.355	**<0.001**
Antibiotic treatment within 24 h postpartum, % (*n*)	54.7 (35)	59.7 (243)	45.9 (85)	**0.008**	0.448	0.228	**0.002**
Parenteral nutrition during catheter dwell time, % (*n*)	50.0 (32)	38.3 (156)	55.1 (102)	**<0.001**	0.076	0.478	**<0.001**

*Data are represented as median (range) unless otherwise specified. For continuous variables, Kruskal–Wallis test was used. *Post hoc* analysis was performed using Mann–Whitney *U* test. For categorical variables, initial and *post hoc* analysis was performed using the Chi-square or Fisher’s exact test. Upon initial testing, *p*-values below 0.05 were considered significant. *Post hoc* testing was significant if *p*-values were below 0.017. Significant *p*-values are depicted in bold*.

Indications for catheter removal varied between catheter location groups (see Table S1 in Supplementary Material). Catheters were still *in situ* at discharge of our department in 28% (181/656) of cases. Follow-up of indications for catheter removal in these infants after discharge was not known. Elective removal of the catheter was performed in 38% (251/656) of cases because they were no longer needed. The remaining 224 catheters with elective removals in our hospital were analyzed. Sepsis (25%; 32/128; *p* < 0.05) and local infiltration (31%; 39/128; *p* < 0.001) were more frequently observed as the cause of non-elective removal in UVCs compared to the other catheters. In FVCs, thrombosis (16%; 3/19; *p* < 0.05) was more often the reason for catheter removal than in the other catheter groups. Catheter occlusion (34%; 26/77; *p* < 0.001) occurred more frequently in PICCs than in the other catheter groups.

**Figure 1:**
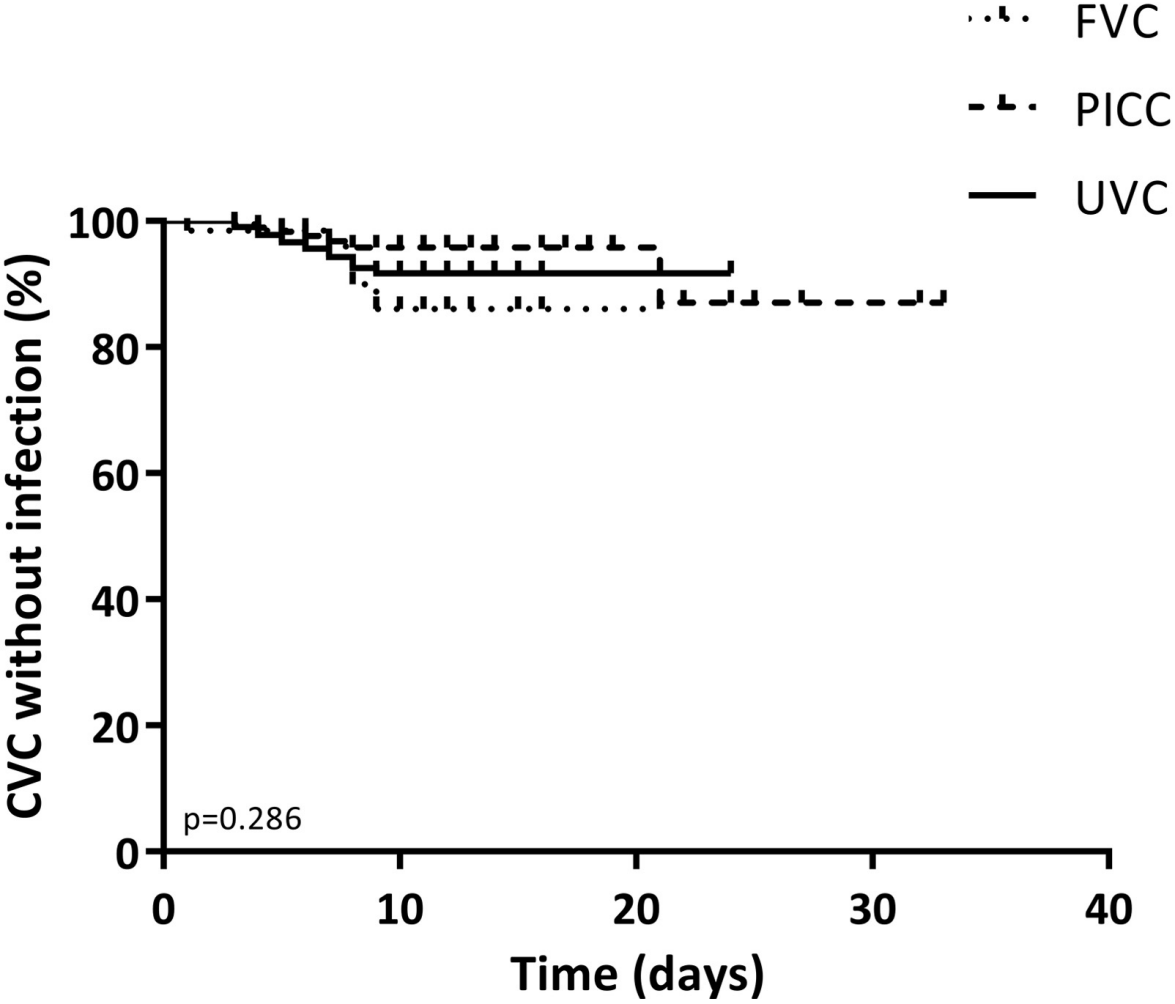
Kaplan–Meier curve of the duration of stay for all catheters by catheter type. The primary end point was the occurrence of a central line-associated bloodstream infection event. Data were censored at elective removal and at patients who were lost to follow-up. *p*-Values below 0.05 were considered significant.

Estimated survival of the three types of catheters regarding CLABSI occurrence is shown in Figure 1. Occurrence of CLABSI did not differ significantly between the three catheter groups (*p* = 0.286).

### Incidence of and Risk Factors for Central Line-Associated Blood Stream Infections

**Figure 2:**
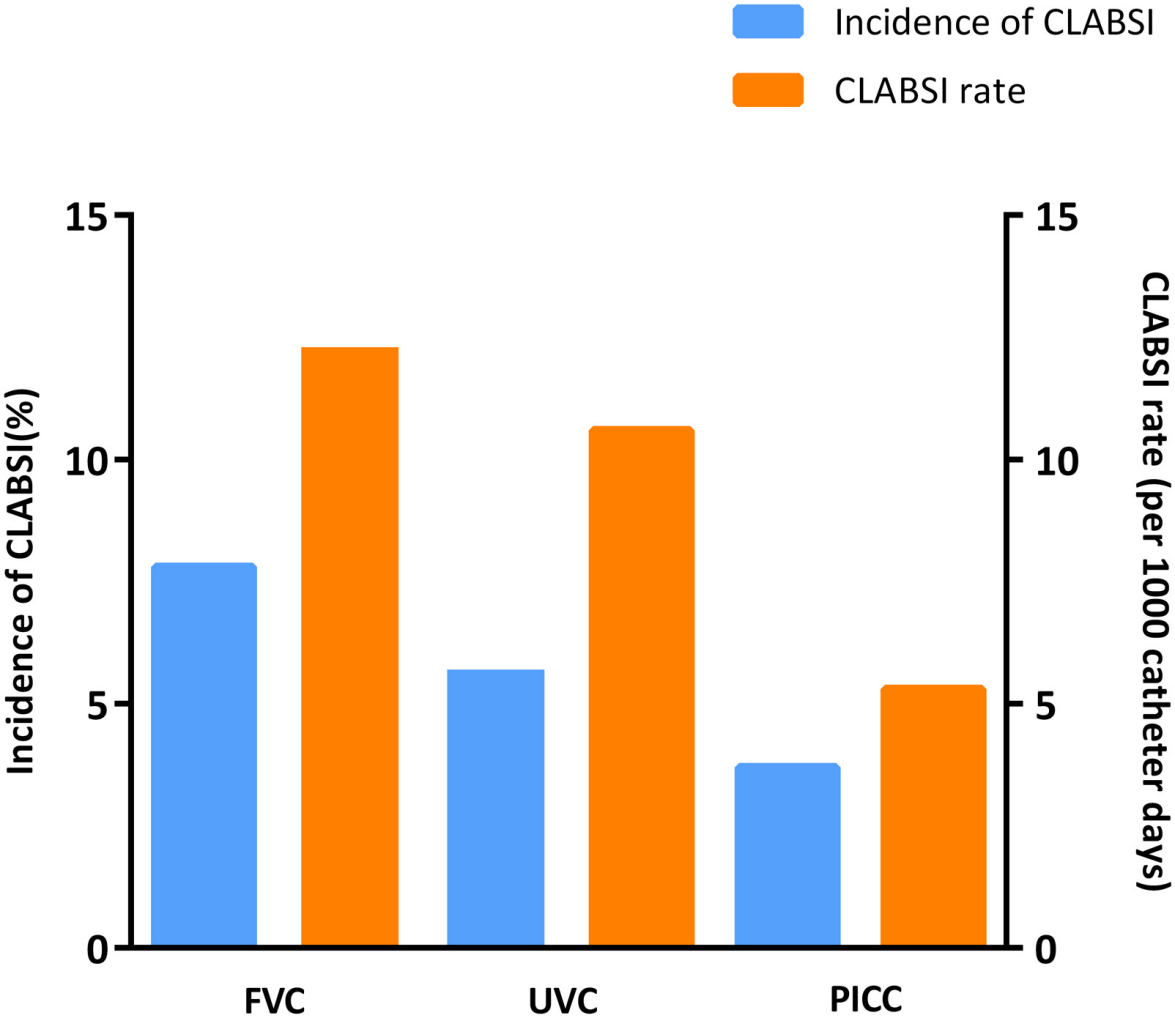
Incidence of central line-associated bloodstream infection (CLABSI) events by catheter type.

In this study, 35 cases (5.3%) of CLABSI were found during the use of 656 catheters or 3,895 catheter-days, which resulted in an overall incidence rate of 9.0 per 1,000 catheter-days. Figure 2 depicts the risks and incidences of CLABSI in the different catheter groups. The risk of CLABSI varied from 4% (7/185) in the PICC group to 8% (5/64) in the FVC group, although differences between the catheter groups were not statistically significant (*p* = 0.42). The CLABSI incidence rate varied from 12.3 per 1,000 catheter-days in FVCs to 10.6 per 1,000 catheter-days in UVCs and 5.3 per 1,000 catheter-days in PICCs.

Coagulase-negative Staphylococci (CoNS; 51%) and *Staphylococcus aureus* (23%) were the most prominent causative organisms of CLABSI. All five CLABSI in the FVC group were caused by CoNS. In the UVC-group, 9 of 23 septic episodes were caused by CoNS (39%), 7 by *S. aureus* (30%), 1 by *E. coli* (4%), 1 by *Bacillus species* (4%), 1 by *Streptococcus agalactiae* (4%), and the other 4 by multiple organisms or unspecified (17%). In the PICC group, four of seven septic episodes were caused by CoNS (57%), one by *S. aureus* (14%), one by *E. coli* (14%), and one by multiple organisms.

On univariate Cox regression analysis, four variables were possibly associated with CLABSI (*p*-values < 0.20). To test the hypothesis that the location of catheter was associated with the risk of CLABSI, we set up a multivariate Cox regression model with location of catheter as dependent variable and all four variables with *p*-values < 0.20 in univariate analysis as independent variables. Male gender [hazard ratio (HR) 2.63], higher birth weight (per 100 g; HR 1.04), and parenteral nutrition during catheter use (HR 2.60) were identified as risk factors. Early antibiotic treatment (HR 0.25) was found to be inversely associated. Table 3 shows these factors including HRs, 95% confidence intervals, and *p*-values. Location of catheter, gestational age at birth, RDS, amount of catheters placed, and prophylactic heparin treatment were not significantly associated with CLABSI.

**Table 3:** Multivariate Cox regression analysis for central line-associated bloodstream infection (CLABSI) events.

Risk factors		CLABSI (*n* = 35)	No CLABSI (*n* = 621)	Hazard ratio (HR)	95% confidence interval (CI) lower	Upper	*p*-Value
Birth weight (g, HR per 100 g)		3,340 (2,120–5,290)	3,125 (807–6,430)	1.04	1.002	1.09	**0.04**
Gender (male), % (*n*)		77 (27)	54 (335)	2.63	1.17	5.90	**0.02**
Location of catheter, % (*n*)	Femoral venous catheter	14 (5)	10 (59)				
	Umbilical venous catheter	66 (23)	62 (384)	1.17	0.44	3.09	0.76
	Peripherally inserted central catheter	20 (7)	29 (178)	0.45	0.14	1.43	0.18
Antibiotic treatment within 24 h postpartum, % (*n*)		40 (14)	56 (349)	0.25	0.12	0.52	**<0.001**
Parenteral nutrition during catheter dwell time, % (*n*)		63 (22)	43 (268)	2.60	1.25	5.41	**0.01**

*Four variables with *p*-values < 0.20 in univariate analysis and location of catheter were included in this multivariate Cox regression model. Data are presented as median (range) unless otherwise specified. HRs including 95% confidence intervals (95% CI) are shown. *p*-Values below 0.05 were considered significant. Significant *p*-values are depicted in bold*.

## Discussion

The overall incidence of CLABSI in our cohort of neonates (≥34 weeks of gestation) with CVCs was 9.0 per 1,000 catheter-days. We did not find a difference in CLABSI risk between FVCs, PICCs, and UVCs.

The reported incidences of CLABSI in neonates admitted to NICUs vary from 0.8 to 18.1 per 1,000 catheter-days (3, 10, 14–19). Variation in reported incidences is probably due to methodological differences between the studies including different cohort characteristics, but may also be attributable to differences in actions to prevent the development of CLABSI between the departments, such as emphasizing hand hygiene, avoidance of certain medications, and using bundles of care. We report the CLABSI incidence in our cohort of neonates born at 34 or more weeks of gestation, while most studies about this topic focus on the total NICU population or only extremely premature infants. The National Healthcare Safety Network reports CLABSI incidences for different birth weight categories separately with a pooled mean varying from 0.8 CLABSI per 1,000 catheter-days in infants >2,500 g to 2.3 CLABSI per 1,000 catheter-days in infants ≤750 g (20). Despite excluding the preterm and primarily smaller infants with the highest risk for infection, the CLABSI incidence in our cohort was high compared to the reported incidence in infants >2,500 g. Low gestational age and hygiene are important factors influencing the incidence of CLABSI. Studies have shown that education of medical staff about hygiene, new disinfectant methods, and the use of specific care bundles for catheter placement can reduce the incidence of CLABSI (15, 17, 21, 22). During the last years, we introduced several evidence-based strategies to reduce the incidence of CLABSI in our NICU. The effect of these actions needs to be evaluated in the future. One contributing factor for the high CLABSI incidence reported in this study might be that the definition of CLABSI used in this study is different from the more strict Center of Disease Control (CDC) criteria used in most other studies. These criteria require at least two positive blood cultures in case of common commensals to fulfill the criteria of CLABSI (23). We used a definition requiring only one positive blood culture in an infant with clinical sepsis. The amount of blood taken from neonates at our department is restricted. Only 1 ml per sample is taken, and blood cultures are usually not repeated when the neonate recovers with antibiotic treatment. Use of the CDC criteria with at least two positive blood cultures would have shown a lower CLABSI incidence, but most likely would have underestimated the true incidence.

In our study, differences in CLABSI incidence between the different catheter types were not significant. The literature is conflicting on what type of CVCs carries the highest infection rate. Shalabi et al. and Arnts et al. found no difference in risk of CLABSI between UVCs and PICCs in neonates born <30 weeks of gestation and in the total NICU population, respectively (7, 8). Other authors compared the risk of CLABSI in infants with three different catheter types, in particular UVCs, PICCs, and other central catheters, including FVCs (3, 9, 10). Yumani et al. advocated that UVCs carry the highest infection rate (10), yet Chien et al. pointed to a higher risk of CLABSI in PICCs and Broviacs compared to UVCs (3). Tsai et al. reported a higher risk of CLABSI in infants with birth weight <1,500 g with FVCs compared to infants with PICCs (11). Most studies that compared catheter types include smaller numbers of term and late preterm infants, and they usually analyze FVCs in a group with other catheters. The only study that compared FVCs with other catheters (UVCs) as a separate catheter type in term infants found a non-significantly higher risk in the FVC group, but their cohort only comprised 19 FVCs (12). It has been suggested previously that femoral catheterization increases the risk of infection, because of proximity to the groin (2, 24). However, if this would be the case, we would have expected mostly Gram-negative microorganisms as causative organisms for CLABSI in the FVC group, whereas only Gram-positive microorganisms (CoNS) were found.

To date, UVCs and PICCs have not been compared to FVCs to this extent before, which is one of the strengths of this study. In addition, this is the largest study to date comparing not only the rates of CLABSI in UVCs and PICCs but also in FVCs in a cohort of term and late preterm neonates.

However, our results have to be interpreted with caution because of the observational study design. As shown in our study, the population of neonates with UVCs or PICCs differed from the population with FVCs. A high proportion of neonates who have a FVC in our department have congenital heart disease (27/64; 42%) sometimes needing therapeutic cardiac catheterization. Hypothetically, different CLABSI incidences in the catheter groups may not be due to the type of catheter, but may be confounded by other clinical factors, such as the underlying clinical condition itself.

Our data support the findings that administration of parenteral nutrition during use of a catheter results in a higher risk of CLABSI, probably due to the lipid constituents in the fluid as found in other studies (25–27). By Cox regression higher birth weight was an independent risk factor for catheter infection, but with a HR of 1.04 this association is weak and seems not clinically relevant. It is in contrast with other reports with higher CLABSI incidences in neonates with lower birth weight (20, 28). However, these studies are performed in populations of neonates with very low birth weight or the total neonatal population in contrast with our cohort of only late preterm and term neonates. We could not find an association between birth weight and CLABSI incidence in a cohort comparable to our cohort. Male gender was associated with a higher risk of CLABSI. The observational study design may lead to bias by indication and it is, therefore, not possible to prove causality. Male gender is associated with a higher neonatal morbidity and mortality hence boys will probably have more or other types of catheters placed which may lead to a higher CLABSI incidence (29, 30).

In our department, treatment with antibiotics during the first 24 h after birth is associated with a lower risk of CLABSI. We could not find this association in prior studies. Antibiotics in the study population were started routinely in neonates with gestational ages below 35 weeks with no clear cause of the prematurity and in the presence of risk factors for infection. In neonates of all gestational ages, antibiotics are started in the presence of risk factors for infection and clinical signs of infection. According to the unit protocol Amoxicillin and Gentamycin are given for 48 h in case of negative cultures. In 12/552 cases (2.2%), culture-proven early onset sepsis was diagnosed, with 11/12 (92%) *Streptococcus agalactiae* as causative organism. Patients who are treated with antibiotics early have a different microbiome (31), and we speculated that they could carry less microorganisms on their skin, decreasing the risk of CLABSI. However, this does not mean that we can promote the use of early antibiotics, because this would, by selection of microorganisms, lead to a larger proportion of resistant causative organisms, although the total number of CLABSI could be lower (32).

In summary, CLABSI remain a problem in the NICU. We did not demonstrate a significant difference in CLABSI risk between three catheter types in the NICU. Several studies conducted on how to decrease the incidence of CLABSI have recently shown that proper education of medical staff, the use of specific care bundles and standardization of protocols can prevent CLABSI (15, 21, 22, 33). More extensive follow-up studies are needed to determine the actual incidences of CLABSI in neonates of all gestational ages, which factors genuinely affect the risk of CLABSI and what methods are the most effective for preventing these events.

## Ethics Statement

The study was approved by the hospital system institutional review board of the Leiden University Medical Center (LUMC). Because of the descriptive study design no written informed consent of the subjects was needed.

## Author Contributions

GD-V was the executive researcher of this study. She performed literature search, data collection, data analysis, data interpretation, and writing and submitting of the report. VB was involved in data analysis, data interpretation, and editing of the report. IP was involved in data collection, data analysis, and writing of the report. EZ was involved in data analysis and interpretation. VS-W and SS were involved in study design, data collection, data interpretation, and editing of the report. AP was involved in study design, data interpretation, and critical revision of the content of the report. EL was the project leader and performed literature search, coordinated data analysis, data interpretation, and writing and editing of the report.

## Conflict of Interest Statement

The authors declare that the research was conducted in the absence of any commercial or financial relationships that could be construed as a potential conflict of interest.

## Abbreviations

CDC, Center of Disease Control; CI, confidence interval; CLABSI, central line-associated bloodstream infection; CoNS, coagulase-negative staphylococci; CVC, central venous catheter; FVC, femoral venous catheter; HR, hazard ratio; LUMC, Leiden University Medical Center; NHSN, National Healthcare Safety Network; NICU, neonatal intensive care unit; PICC, peripherally inserted central catheter; RDS, respiratory distress syndrome; SGA, small for gestational age; UVC, umbilical venous catheter.
