# Supplementary material for: Bloodstream Infection Incidence of Different Central Venous Catheters in Neonates: A Descriptive Cohort Study
Source: Front Pediatr. 2017 Jun 20;5:142. doi: 10.3389/fped.2017.00142 (PMC5477168; doi:10.3389/fped.2017.00142)
Supplement: Supplementary file 1 [file Table_1.DOCX]

| Reason for catheter removal | FVC *(n=64)* | UVC *(n=407)* | PICC *(n=185)* |
| --- | --- | --- | --- |
| Suspected sepsis | 4 | 32 | 7 |
| Phlebitis | 1 | 0 | 10 |
| Local infiltration | 1 | 39 | 7 |
| Leakage | 4 | 15 | 6 |
| Occlusion | 4 | 4 | 26 |
| Thrombosis | 3 | 3 | 2 |
| Malposition | 1 | 32 | 11 |
| Unknown | 1 | 3 | 8 |
| No longer needed | 24 | 187 | 40 |
| Still in situ at discharge | 21 | 92 | 68 |
